# Multifactorial anticancer effects of digalloyl-resveratrol encompass apoptosis, cell-cycle arrest, and inhibition of lymphendothelial gap formation *in vitro*

**DOI:** 10.1038/sj.bjc.6605656

**Published:** 2010-04-27

**Authors:** S Madlener, P Saiko, C Vonach, K Viola, N Huttary, N Stark, R Popescu, M Gridling, N T-P Vo, I Herbacek, A Davidovits, B Giessrigl, S Venkateswarlu, S Geleff, W Jäger, M Grusch, D Kerjaschki, W Mikulits, T Golakoti, M Fritzer-Szekeres, T Szekeres, G Krupitza

**Affiliations:** 1 Institute of Clinical Pathology, Medical University of Vienna, Vienna, Austria; 2 Clinical Institute of Medical and Chemical Laboratory Diagnostics, Medical University of Vienna, Vienna, Austria; 3 Department of Clinical Pharmacy and Diagnostics, University of Vienna, Vienna, Austria; 4 Department of Pharmacognosy, University of Vienna, Vienna, Austria; 5 Department of Medicine I, Institute of Cancer Research, Medical University of Vienna, Vienna, Austria; 6 Laila Impex R&D Center Unit I, Vijayawada, Andhra Pradesh, India

**Keywords:** digalloyl-resveratrol, anti-neoplastic, Cdc25A, ribonucleotide reductase, lymphendothelial retraction

## Abstract

**Background:**

Digalloyl-resveratrol (di-GA) is a synthetic compound aimed to combine the biological effects of the plant polyhydroxy phenols gallic acid and resveratrol, which are both radical scavengers and cyclooxygenase inhibitors exhibiting anticancer activity. Their broad spectrum of activities may probably be due to adjacent free hydroxyl groups.

**Methods:**

Protein activation and expression were analysed by western blotting, deoxyribonucleoside triphosphate levels by HPLC, ribonucleotide reductase activity by ^14^C-cytidine incorporation into nascent DNA and cell-cycle distribution by FACS. Apoptosis was measured by Hoechst 33258/propidium iodide double staining of nuclear chromatin and the formation of gaps into the lymphendothelial barrier in a three-dimensional co-culture model consisting of MCF-7 tumour cell spheroids and human lymphendothelial monolayers.

**Results:**

In HL-60 leukaemia cells, di-GA activated caspase 3 and dose-dependently induced apoptosis. It further inhibited cell-cycle progression in the G1 phase by four different mechanisms: rapid downregulation of cyclin D1, induction of Chk2 with simultaneous downregulation of Cdc25A, induction of the Cdk-inhibitor p21^Cip/Waf^ and inhibition of ribonucleotide reductase activity resulting in reduced dCTP and dTTP levels. Furthermore, di-GA inhibited the generation of lymphendothelial gaps by cancer cell spheroid-secreted lipoxygenase metabolites. Lymphendothelial gaps, adjacent to tumour bulks, can be considered as gates facilitating metastatic spread.

**Conclusion:**

These data show that di-GA exhibits three distinct anticancer activities: induction of apoptosis, cell-cycle arrest and disruption of cancer cell-induced lymphendothelial disintegration.

Digalloyl-resveratrol (di-GA) is a synthetic ester of the phytoalexin resveratrol (3,4′,5-trihydroxystilbene; RV) and the polyhydroxy phenolic compound gallic acid (3,4,5-trihydroxybenzoic acid; GA) (Figure 1). Gallic acid can be found in various natural products, such as green tea, pineapples, bananas, apple peels, red and white wine (Sun *et al*, 2002; De Beer *et al*, 2003; Wolfe *et al*, 2003). Resveratrol is a constituent of red wine and grapes. Both compounds are proposed to contribute to the ‘French Paradox’, a phenomenon of significantly lower (40%) heart infarction incidence in the French population, when compared with other European countries or the United States (Richard, 1987; Renaud and De Lorgeril, 1992; Constant, 1997). Gallic acid and RV were also described as excellent free radical scavengers (Inoue *et al*, 1994; Isuzugawa *et al*, 2001; Kawada *et al*, 2001; Salucci *et al*, 2002; Sohi *et al*, 2003; Horvath *et al*, 2005) and as inducers of differentiation and programmed cell death in a variety of tumour cell lines. Other beneficial properties of GA-containing fruit extracts include anti-diabetic and anti-angiogenic effects (Liu *et al*, 2005; Sridhar *et al*, 2005). Gallic acid is also present at high concentrations in gallnuts (name), which are proliferations of plant leaves that become elicited by gall wasp exudates to build up a hatchery for their larvae. Thus, the secretion of gall wasps stimulates plant cell growth and overrules homeostasis of the affected leaf area – this is similar to tumour outgrowth. In turn, the plant produces GA, which seems to combat the improper growth signals and re-establishes cell-cycle control. This could at least explain why gallnuts are rich in GA and that gallnuts do not grow beyond a certain size. This cytostatic property of GA – which is amplified in di-GA – seems to be one of the cancer-protective principles of a variety of fruits and this could also be developed for adjuvant therapy.

Gallnuts are not used in modern western medicine, but they were mentioned in the first book of ‘De Materia Medica’ ascribed to Pedanios Dioscurides (the ‘Vienna Dioscurides’, Austrian National Library, which was written in the sixth century in Konstantinopolis, East Roman Empire). Interestingly, this manuscript claims that gallnuts ‘stop the growth of proliferating tissue’. Other studies showed that RV and GA are effective inhibitors of the enzyme ribonucleotide reductase (RR; EC1.17.4.1) (Fontecave *et al*, 1998; Madlener *et al*, 2007). Ribonucleotide reductase is significantly upregulated in malignant cells compared to non-malignant cells. This enzyme catalyses the rate-limiting step of *de novo* DNA synthesis, which is the reduction of ribonucleotides into the corresponding deoxyribonucleoside triphosphates (dNTPs). This qualifies RR as an excellent target for cancer chemotherapy.

Apart from being a radical scavenger, the multifactorial effects of GA encompass also the inhibition of cyclooxygenases (COXs) and of lipoxygenases (LOXs). Tumours express high levels of COX-2 and 12-LOX (Nie *et al*, 2003; Pidgeon *et al*, 2003; Nassar *et al*, 2007), which metabolise arachidonic acid to prostanoids and to hydroxyeicosatetraenoic acids (12(*S*)-HETE), respectively (Marks *et al*, 2000). Certain HETEs function as inter- and intracellular messengers and cause the repulsion of endothelial cells thereby forming gaps in the endothelial cell layer (Ohigashi *et al*, 1989; Nakamori *et al*, 1997; Uchide *et al*, 2007). Further, these gaps may serve as entry ports for adjacent tumour cells into the lymphatic system. Thus, we hypothesised that GA (and di-GA) may inhibit lymphendothelial gap formation. Here we examine the effects of di-GA on apoptosis, cell-cycle progression and lymphendothelial gap formation.

## Materials and Methods

### Chemicals

Nordihydroguaiaretic acid (NDGA) was from Cayman Chemical (Ann Arbor, MI, USA); and aspirin, mannitol, probucol, GA and RV were from Sigma-Aldrich (Vienna, Austria). Catalase and carboxy-PTIO were from Calbiochem-Merck Biosciences (Nottingham, UK). Berberine chloride dihydrate (purity 98.92%) was from Phytolab (Vestenbergsgreuth, Germany). Experimental stock solutions (in DMSO) were prepared always fresh.

Mouse monoclonal anti-Cdc25A (F-6) Cat. No. 7389; anti-PARP-1 (F-2) Cat. No. sc-8007; anti-cyclin D1 (M-20) Cat. No. sc-718; anti-cyclin E (M20) Cat. No. sc-481 and anti-p21^Cip/Waf^ (C-19) Cat. No. sc-397 antibodies were from Santa Cruz Biotechnology Inc. (Heidelberg, Germany). Polyclonal anti-phospho-Cdc25A (Ser17) Cat. No. ab18321 antibody was from Abcam (Cambridge, UK); and monoclonal anti-p34^Cdc2^ Cat. No. C3085 and anti-*β*-actin (AC15) Cat. No. A5441 antibodies were from Sigma-Aldrich. Rabbit monoclonal anti-cleaved caspase 3 (CPP32) clone C92-605 Cat. No. 58404 antibody was from Research Diagnostics Inc. (Flanders, NJ, USA). Polyclonal anti-MEK 1/2 Cat. No. 9122; anti-phospho-MEK 1/2 (Ser217/221) Cat. No. 9121 m; anti-phospho-Chk2 (Thr68) Cat. No. 2661; anti-Chk2 Cat. No. 2662 and rabbit monoclonal anti-p44/42 MAP Kinase (137F5) Cat. No. 4695; anti-phospho-Cdc2 (Tyr15) Cat. No. 4539 and mouse monoclonal anti-phospho-p44/42 MAPK (Thr202/Tyr204) (E10) Cat. No. 9106 antibodies were from Cell Signaling Technology Inc. (Danvers, MA, USA). Anti-mouse IgG was from Dako (Vienna, Austria). Anti-rabbit IgG and Amersham ECL – high-performance chemiluminescence film – were from GE Healthcare (Vienna, Austria).

### Cell culture

HL-60 human promyelocytic cells were purchased from ATCC (Wesel, Germany). Cells were grown in RPMI-1640 medium supplemented with 10% heat-inactivated fetal calf serum, 1% L-glutamine and 1% penicillin/streptomycin. MCF-7 cells were grown in McCoy 5A medium containing 10% fetal calf serum and 1% penicillin/streptomycin. Human normal lung fibroblasts (HLF) were a generous gift of the Cancer Research Institute of the Medical University of Vienna and were grown in RPMI medium containing 10% fetal calf serum and 1% penicillin/streptomycin. All media, supplements and G418 were obtained from Life Technologies (Lofer, Austria).

Human dermal microvascular endothelial cells (C-12260) were purchased from PromoCell (Heidelberg, Germany). To obtain a population of highly enriched lymphendothelial cells (LECs) dermal microvascular endothelial cells were sorted with polyclonal rabbit anti-human podoplanin antibody and sheep anti-rabbit dynabeads (M-280; Dynal 11203; Invitrogen, Lofer, Austria). Subsequently, residual cells were sorted with anti-CD31 (Dynal 11128). Incubations were performed at 4 °C for 30 min. Such isolated LECs were stable transfected with telomerase cDNA and then maintained in EGM2 Mv medium (EBM2-based medium CC3156 and supplement CC4147; Lonza, Walkersville, MD, USA) and G-418 (Schoppmann *et al*, 2004). All cell types were kept in humidified atmosphere containing 5% CO_2_ at 37 °C.

### Proliferation inhibition assay

HL-60 cells were seeded in T-25 tissue culture flasks at a concentration of 1 × 10^5^ per ml and incubated with increasing concentrations of di-GA (2.5, 5, 7.5, 10 and 40 *μ*M). Cell numbers and I_p_C_50_ values were determined after 24 and 48 h using a CC-108 microcellcounter (Sysmex, Kobe, Japan).

### Determination of deoxyribonucleoside triphosphates

The extraction of cellular dNTPs was performed according to a method described previously (Garrett and Santi, 1979). HL-60 cells (7 × 10^7^) were incubated with 5, 10 and 40 *μ*M di-GA for 24 h. Then, 1 × 10^8^ were centrifuged at 1800 r.p.m. and resuspended in 100 *μ*l phosphate-buffered saline (PBS) and extracted with 10 *μ*l trichloroacetic acid. The lysate was rested on ice and neutralised by adding 1.5 vol of freon containing 500 *μ*M tri-n-octylamin. Afterwards the lysate was centrifuged (15 000 r.p.m. for 4 min) and the supernatant was used for periodation (100 *μ*l extract +30 *μ*l 4 M methylamine (pH 7.5)+10 *μ*l periodat). Aliquots (120 *μ*l) of each sample were analysed using a Merck ‘La Chrom’ HPLC-system equipped with D-7000 interface, L-7100 pump, L-7200 autosampler and L-7400 UV-detector. Detection time was set at 80 min, the detector operated on 280 nm for 40 min and then switched to 260 nm for another 40 min. Samples were eluted with a 3.2 M ammonium phosphate buffer, pH 3.6 (pH adjusted by addition of 3.2 M H_3_PO_4_), containing 20 mol l^−1^ acetonitrile using a 4.6 × 250 mm Partisil 10 SAX column (Whatman Ltd., Kent, UK). Separation was performed at constant ambient temperature and at a flow rate of 2 ml min^−1^. The concentrations of each dNTP of the experimental samples were then calculated as percent of total area under the control curves. Chemicals were from Sigma-Aldrich and of highest available quality.

### Hoechst 33258 and propidium iodide double staining

The vitality staining was performed according to a protocol described before (Grusch *et al*, 2002). HL-60 cells (0.4 × 10^6^ per ml) were seeded in T-25 tissue culture flasks and exposed to increasing concentrations of di-GA (2.5, 5, 7.5, 10 and 40 *μ*M) for 24 h. Hoechst 33258 and propidium iodide were purchased from Sigma-Aldrich and added directly to the cells at final concentrations of 5 and 2 *μ*g/ml, respectively. After 60 min of incubation at 37 °C, we examined cells with a Zeiss Axiovert fluorescence microscope and a DAPI filter (Carl Zeiss, Jena, Germany). Cells were photographed and analysed by visual examination (not by FACS). This method allows to distinguish between early apoptosis, late apoptosis and necrosis. Cells were judged according to their nuclear morphology and the disintegration of their cell membranes, which is indicated by propidium iodide uptake.

### Cell-cycle distribution analysis

HL-60 cells (0.4 × 10^6^ per ml) were seeded in T-25 tissue culture flasks and incubated with 2.5, 5, 10 and 40 *μ*M di-GA. After 24 h, cells were harvested, washed with 5 ml cold PBS, centrifuged (600 r.p.m. for 5 min) and resuspended and fixed in 3 ml ethanol (70%) at 4 °C for 30 min. After two further washing steps with cold PBS, RNAse A and propidium iodide were added to a final concentration of 50 *μ*g ml^−1^ each and incubated at 4 °C for 60 min before analysis on a FACSCalibur flow cytometer (BD Biosciences, San Jose, CA, USA). The cell-cycle distribution was calculated with ModFit LT software (Verity Software House, Topsham, ME, USA).

### Determination of RR *in situ* activity

Exponentially growing HL-60 cells (5 × 10^5^) were incubated with 1, 2.5 and 5 *μ*M di-GA for 24 h at 37 °C in a humidified atmosphere containing 5% CO_2_ to assess changes in RR *in situ* activity. Then, cells were pulsed with ^14^C-cytidine (Sigma-Aldrich; 3 *μ*l in a 5 ml cell suspension) at 37 °C for 30 min, collected by centrifugation (1200 r.p.m. for 5 min), washed twice with PBS and processed to extract total genomic DNA. Thereafter, the radioactivity, which became incorporated into genomic DNA, was measured.

### Western blotting

HL-60 cells (1.5 × 10^7^ cells) were seeded into T-75 tissue culture flasks and incubated with 10 *μ*M di-GA for 0.5, 2, 4, 8 and 24 h. Then, 1 × 10^6^ cells were harvested (per experimental point), washed twice with cold PBS, centrifuged at 1000 r.p.m. for 5 min and lysed in a buffer containing 150 mM NaCl, 50 mM Tris (pH 8.0), 1% Triton X-100, 1 mM phenylmethylsulfonyl fluoride and protease inhibitor cocktail (from a × 100 stock; Sigma-Aldrich). The lysates were centrifuged at 4 °C for 20 min (12 000 r.p.m.) and supernatants stored at −20  °C until further analysis. Equal amounts of protein samples were separated by polyacrylamide gel electrophoresis and electroblotted onto PVDF membranes (Hybond, GE Healthcare) at 4 °C overnight. Equal sample loading was controlled by staining membranes with Poinceau S (Sigma-Aldrich). After washing with PBS/0.5% Tween 20 (PBS/T) (pH 7.2) or TBS/0.1% Tween 20 (TBS/T) (pH 7.6), membranes were blocked for 1 h in blocking solution (5% non-fat dry milk in PBS/T or in TBS/T). The membranes were incubated with the first antibody (in blocking solution, dilution 1 : 500–1 : 1000) by gently rocking at 4 °C overnight. Thereafter, the membranes were washed with PBS or TBS and further incubated with the second antibody (peroxidase-conjugated goat anti-rabbit IgG or anti-mouse IgG, dilution 1 : 2000–1 : 5000 in PBS/T or TBS/T) for 12 h. Chemoluminescence was developed by the ECL detection kit and the exposure of membranes to Amersham Hyperfilms (GE Healthcare).

### MCF-7 spheroid generation

1.2 g of autoclaved methyl cellulose (M-0512; Sigma-Aldrich) was resuspended in 100 ml prewarmed McCoy 5A medium (Life Technologies; 1.2% stock concentration), stirred until the solution turned clear and centrifuged at 4000 r.p.m. (swing out rotor) for 2 h to pellet undesired debris. Then, 1 × 10^5^ MCF-7 cells were transferred to 15 ml McCoy 5A medium containing 0.24% methyl cellulose (final concentration). 150 *μ*l (containing ∼1 × 10^3^ cells) was transferred to each well of a round bottom microtitre plate (96-well) to allow spheroid formation. Cells were allowed to aggregate and grow for 2 days, and then spheroids were sufficiently dense for further manipulations. MCF-7 spheroids had an average diameter of ∼300 *μ*m.

### MCF-7 spheroid/LEC monolayer co-cultivation

LECs were seeded in EGM2 MV medium on 24-well plates and allowed to grow for 2–3 days until confluence. Then, LECs were stained with cytotracker green (concentration 2 *μ*g ml^−1^ final concentration, Molecular Probes-C2925, Invitrogen) at 37 °C for 90 min and subsequently rinsed thoroughly. Thereafter, MCF-7 spheroids were washed in EGM2 MV medium to rid off methyl cellulose, and 12 spheroids were carefully transferred using wide bore yellow tips to each well containing LECs.

For those experiments in which inhibitors were used, the indicated inhibitor concentrations (final concentrations) were applied to the spheroids 30 min prior addition of the spheroids to the LEC layers.

### Analysis of gap formation

LEC areas with spheroids on top were photographed using an FITC filter, which was used to visualise cytotracker (green)-stained LECs underneath the spheroids. Axiovert software (Carl Zeiss) facilitated to measure the gap areas within the LEC layers.

### Statistical calculations

Dose–response curves were calculated using the Prism 4.03 software package (GraphPad, San Diego, CA, USA) and statistical significance was determined by two-tailed paired *t*-test (significance *P*<0.05).

## Results

Quite a few studies on GA and its derivatives, RV and RV analogues were performed in human leukaemia cells (Saiko *et al*, 2008), because these cells are very sensitive to drugs and therefore advantageous to test the efficacy of novel anticancer compounds. HL-60 cells are particularly useful to discriminate the nuclear morphology of necrotic and apoptotic cells (Grusch *et al*, 2002) and hence, we used HL-60 cells to study di-GA facilitating the comparability of our results with published data of other GA and RV analogues.

### Di-GA induces caspase 3 and apoptosis

**Figure 2:**
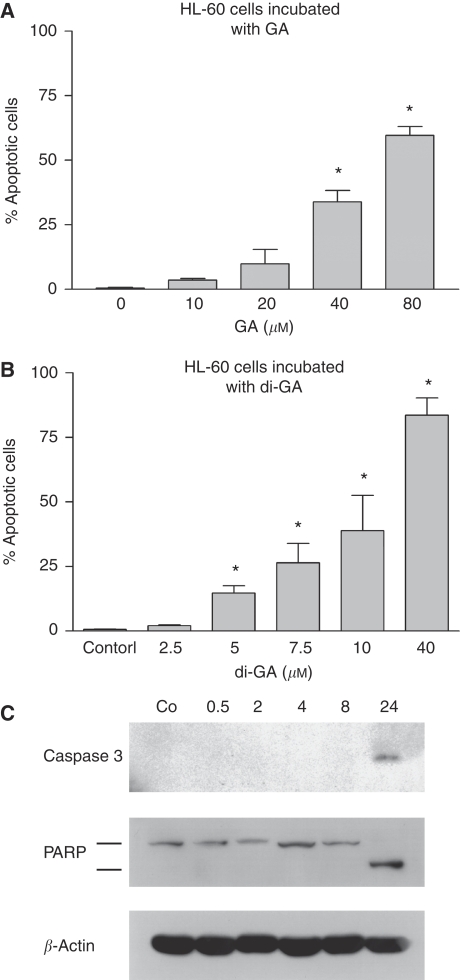
Induction of apoptosis by (**A**) GA and (**B**) di-GA in HL-60 cells. Cells were incubated with increasing concentrations of drugs for 24 h, and then double stained with Hoechst 33258 and propidium iodide. Afterwards cells were examined under the microscope with UV light connected to a DAPI filter. Nuclei with a morphological phenotype indicating apoptosis were counted and percentages of apoptotic cells were calculated. Experiments were conducted in triplicate. Error bars indicate s.e.m., asterisks significance (*P*<0.05). (**C**) Activation of caspase 3 and cleavage of PARP on treatment with di-GA. Logarithmically growing HL-60 cells were incubated with 10 *μ*M di-GA for 0.5, 2, 4, 8 and 24 h. Afterwards cells were lysed and protein expression was analysed by western blotting. The anti-caspase 3 antibody recognises only the cleaved peptide indicating its activation. Anti-PARP antibody recognises the full-length form (116 kDa) and the signature-type cleaved product (85 kDa) that is generated by active caspase 3. The antibody against *β*-actin was used to monitor equal sample loading.

The pro-apoptotic potential of naturally occurring GA was compared to that of synthetic di-GA by incubating HL-60 promyelocytic leukaemia cells to both agents (Figure 2A and B). Increasing concentrations of GA (10, 20, 40 and 80 *μ*M) elicited 4, 10, 34 and 60% apoptosis, respectively. Because the di-GA molecule contains two galloyl residues (as compared to just one gallic acid molecule of GA) we expected that half of the di-GA concentrations would induce similar apoptosis rates as the tested GA concentrations. However, 5, 10 and 40 *μ*M di-GA (to compare it to 10, 20 and 80 *μ*M GA, see above) triggered 12, 39 and 84% apoptosis, respectively. In an earlier study, we showed that 25 and 50 *μ*M RV induced ∼18 and 45% apoptosis in HL-60 cells, respectively (Horvath *et al*, 2006). Therefore, the apoptotic efficiency of di-GA is the sum of the apoptotic properties of 2 × GA plus RV. Apoptosis correlated with the activation of caspase 3 and with the signature type cleavage of PARP into an 85 kDa fragment (Figure 2C). Digalloyl-resveratrol did not induce significant numbers of necrotic cells even at high concentrations (data not shown). The data suggest that di-GA is a potent inducer of apoptosis and significantly more effective than GA alone.

### Di-GA inhibits G1-S transition

**Figure 3:**
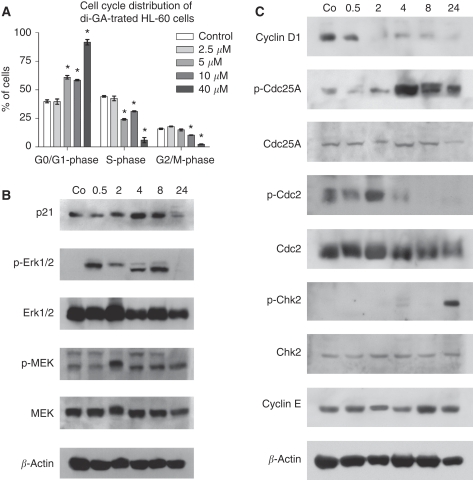
Effect of di-GA on the cell cycle of HL-60 cells. (**A**) Logarithmically growing HL-60 cells were incubated with increasing concentrations of di-GA for 24 h and then subjected to FACS analysis. Experiments were conducted in triplicate. Error bars indicate s.e.m., asterisks significance (*P*<0.05). HL-60 cells were incubated with 10 *μ*M di-GA for 0.5, 2, 4, 8 and 24 h, lysed, and the (**B**) expression of p21^Cip/Waf^, the phosphorylation of threonine202/tyrosine204-Erk1/2 (p-Erk1/2) and serine217/221-MEK1/2 (p-MEK), and (**C**) phosphorylation of threonine68-Chk2 (p-Chk2), serine17-Cdc25A (p-Cdc25A), tyrosine15-Cdc2 (p-Cdc2), and the protein levels of cyclin D1, E were analysed by western blotting. *β*-Actin served as loading control.

HL-60 cells were exposed to increasing concentrations of GA and di-GA and the cell numbers were measured after 24 and 48 h. The percentages of proliferation inhibition were calculated at both time points. Those concentrations that inhibited 50% proliferation (I_p_C_50_) are shown in Table 1. Digalloyl-resveratrol inhibited proliferation 7–10 times more efficiently than GA during the tested time period. Inhibition of cell proliferation was due to a dose-dependent cell-cycle block in G1 (Figure 3A).

### Di-GA modulates mitogenic signalling and the expression of cell-cycle regulators

We next examined the levels of the cell-cycle inhibitor p21^Cip/Waf^, which is known to inhibit Cdk2 by blocking its interaction with cyclin E (Jeon *et al*, 2007). p21^Cip/Waf^ was induced within 4 h (Figure 3B), which was independent of p53, because HL-60 cells are p53 negative (Biroccio *et al*, 1999). Phosphorylation of Erk1 and MEK, which is indicative for their activation, preceded the increase in p21^Cip/Waf^ levels. This is consistent with previous reports that MEK-Erk signalling upregulates p21^Cip/Waf^ (Facchinetti *et al*, 2004; Park *et al*, 2004; Perez-Pinera *et al*, 2006). Phosphorylation of Erk2 (the lower band occurring after 4 and 8 h) was simultaneous to p21^Cip/Waf^ upregulation. Next, we investigated whether the expression of the G1-specific cell-cycle regulators Cdc25A, cyclin D1 and cyclin E was altered by di-GA treatment (10 *μ*M). Western blot analyses showed that cyclin D1 expression decreased after 2 h and remained suppressed, whereas cyclin E expression persisted (Figure 3C). Cyclin D1 is required for the activation of Cdk4 and Cdk6 (Lingfei *et al*, 1998; Alao, 2007), which altogether is controlled by Cdc25A (Iavarone and Massague, 1997). Digalloyl-resveratrol strongly induced serine 17 (Ser17) phosphorylation of Cdc25A after 4 h. Phosphorylation of Ser17-Cdc25A was shown to stabilise this phosphatase at a high activity status specifically in the M phase (Mailand *et al*, 2002), thereby de-phosphorylating and activating its target Cdk1 (Cdc2). This is mandatory for the transit through the G2-M phase (Karlsson-Rosenthal and Millar, 2006). Hence, Cdc25A controls not only the G1-S, but also the G2-M phase. Indeed, di-GA caused the de-phosphorylation of Tyr15-Cdc2 indicating that cells entered the mitotic phase. FACS analysis confirmed that 40 *μ*M di-GA allowed ∼90% of the cells to pass through S and M phase (likely due to Cdc25A activity) but accumulated in the subsequent G1 phase because cyclin D1 was repressed. Finally, Cdc25A protein level decreased after 24 h. This was paralleled by Chk2 activation (indicated by its phosphorylation at Thr68), presumably due to replicatory stress. Chk2 targets Cdc25A for proteolytic degradation (Karlsson-Rosenthal and Millar, 2006). In summary, the data suggest that di-GA inhibits cell proliferation by disturbing orchestrated mitogenic signalling.

### Di-GA inhibits RR

Gallic acid is a radical scavenger (Whang *et al*, 2005) and inhibits RR through chelating the tyrosyl radical required for RR activity (Madlener *et al*, 2007). Ribonucleotide reductase is the rate-limiting enzyme for nucleotide metabolism necessary for DNA synthesis during cell division.

**Figure 4:**
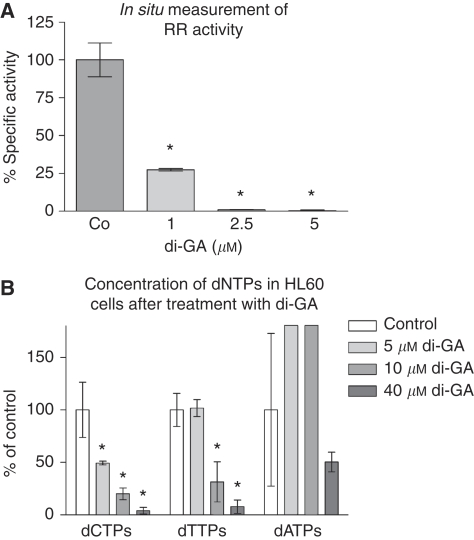
(**A**) Measurement of the *in situ* effect of di-GA on ribonucleotide reductase (RR) activity. HL-60 cells were incubated with 1, 2.5 and 5 *μ*M di-GA for 24 h at 37 °C in a humidified atmosphere containing 5% CO_2_ to assess changes in RR *in situ* activity. Then, cells were pulsed with ^14^C-cytidine (Sigma-Aldrich; 3 *μ*l in a 5 ml cell suspension) for 30 min at 37 °C. Afterwards the cells were collected and the radioactivity that became incorporated into genomic DNA was measured. (**B**) Effect of di-GA on intracellular dNTP pools in HL-60 cells. HL-60 cells were incubated with 5, 10 and 40 *μ*M di-GA for 24 h. Then the cells were prepared for HPLC analysis and the dNTP levels were determined according to the protocol described in the ‘Materials and methods’ section. Experiments were conducted in triplicate. Error bars indicate s.e.m., asterisks significance (*P*<0.05).

Hence, RR activity was investigated by an assay that measures the incorporation of ^14^C-cytidin into genomic DNA. Figure 4A shows that ^14^C-cytidin incorporation into genomic DNA decreased with increasing di-GA concentration. Further, RR activity was fully blocked on treatment with 5 *μ*M di-GA. At this concentration the dCTP level (but not dTTP and dATP) dropped significantly (Figure 4B). In HT29 colon carcinoma cells, a similar effect of di-GA on RR activity, dCTP, dTTP and dATP levels was observed (Bernhaus *et al*, 2009).

### Di-GA inhibits lymphendothelial gap formation induced by co-cultivated tumour cell spheroids

**Figure 5:**
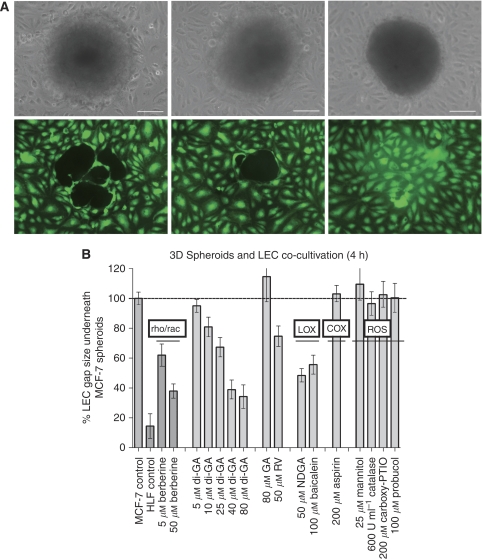
Effect of di-GA on MCF-7 spheroid-induced gap formation in lymphendothelial cell monolayers. (**A**) LEC monolayers that were exposed to MCF-7 spheroid (left side panels), MCF-7 spheroid treated with 40 *μ*M di-GA (middle panels) and HLF spheroid (right side panels). Upper panels are phase-contrast micrographs showing the respective spheroids, the panels below show the identical power fields using FITC filter and exhibit green stained LECs underneath the respective spheroids. Bars in the lower right corners of upper panels indicate 100 *μ*M. (**B**) MCF-7 tumor spheroids were preincubated with solvent (control), or 5 and 50 *μ*M berberine; 5, 10, 25, 40 and 80 *μ*M di-GA; 80 *μ*M GA; 50 *μ*M RV; 50 *μ*M NDGA; 100 *μ*M baicalein; 200 *μ*M aspirin; 25 *μ*M mannitol; 600 U ml^−1^ catalase; 200 *μ*M carboxy-PTIO and 100 *μ*M probucol, and then placed on top of cytotracker stained LEC monolayers that were also treated with respective agents for 4 h. Then, the size of the gaps that were formed in the LEC monolayers by MCF-7 spheroids (through repulsion of LECs) was measured using an inverted microscope connected to an FITC filter and equipped with Axiovision 4.5 software (Carl Zeiss). As negative controls normal human lung fibroblast (HLF) spheroids were used. Rho/rac (small GTPases regulating cell migration), LOX (lipoxygenase), COX (cyclooxygenases) and ROS (reactive oxygen species) indicate which mechanisms and phenomena are inhibited by the respective agents. Experiments were conducted in triplicate, and the underneath areas of at least 12 spheroids were analysed. Error bars indicate s.e.m., asterisks significance (*P*<0.05).

Leukocytes trespass basal membranes and trans-migrate tissues and endothelia as part of their normal physiological function and are therefore, *a priori* ‘invasive’. Hence, HL-60 leukaemia cells are inappropriate to study the pathological invasiveness of cancer cells and the anti-invasive/anti-metastatic potential of di-GA. In contrast, solid tumours acquire an invasive potential in course of cancer progression and this particular cancer cell property has to be studied and combated. We developed a novel bulk invasion assay to establish an *in vitro* model resembling the pathologic situation of ductal breast cancer cells invading the lymphatic vasculature and to recapitulate the mechanism of metastasis (Ohigashi *et al*, 1989; Nakamori *et al*, 1997; Uchide *et al*, 2007). For this, telomerase immortalised human LECs were grown to confluent monolayers and MCF-7 tumour spheroids (average diameter ∼300 *μ*m, containing ∼4000 cells) were placed on top to mimic tumour intrusion into lymphatics. Lymphendothelial cells were pre-labelled with cyto-tracker (green) immediately before co-cultivation, to monitor presence or absence of LECs underneath tumour spheroids (Figure 5A). Normal HLF spheroids served as negative controls, because these primary cells with limited lifespan (Hayflick limit) are non-malignant and do not invade blood or lymphatic vasculature. After 4 h of co-cultivation, gaps formed underneath >99% of the MCF-7 tumour spheroids (gap area was on average ∼1.15 × 10^5^ *μ*m^2^) whereas no or only small gaps were formed underneath normal lung fibroblasts. The gap size area was measured underneath at least 12 spheroids and in triplicate experiments. These gaps resemble entry ports for cancer cell bulks invading the lymphatic system, which is now widely accepted to be a route for the spreading of certain cancers (Alitalo *et al*, 2005; Oliver and Alitalo, 2005; Sipos *et al*, 2005).

Di-GA inhibited gap formation dose-dependently and maximally by >60% (Figure 5B). We have evidence (time-laps movies; data not shown) that gap formation is caused by LEC migration. Berberine was reported to inhibit cell migration and invasion of SCC-4 tongue squamous cancer cells (Ho *et al*, 2009) and HONE1 nasopharyngeal cancer cells (Tsang *et al*, 2009). The chemical structure of berberine is reminiscent to parts of di-GA and for control reasons we tested whether berberine had an effect on MCF-7-induced LEC behaviour. Berberine dose-dependently inhibited gap formation and this confirmed that the assay was functional and responded according to prediction.

Primary cancers and also MCF-7 breast cancer cells express elevated levels of LOXs, which metabolise arachidonic acid to HETEs (Marks *et al*, 2000; Nie *et al*, 2003; Kudryavtsev *et al*, 2005). The migration of endothelial cells was shown to be mediated by LOXs generating 12(*S*)-HETE (Ohigashi *et al*, 1989; Nakamori *et al*, 1997; Uchide *et al*, 2007). 12(*S*)-HETE functions as inter- and intracellular messenger and causes the retraction of endothelial cells, thereby forming gaps into the confluent cell layer. The 12/15-LOX inhibitors baicalein (100 *μ*M) and NDGA (50 *μ*M) reduced the area of MCF-7 spheroid-induced gaps in the LEC monolayers by ∼50 and 60%, respectively. Derivatives of GA are also known to inhibit HETE generating LOXs, and prostanoids generating COXs (Christow *et al*, 1991; Ha *et al*, 2004; Kim *et al*, 2006). However, because aspirin had no effect on gap formation (Figure 5B) the contribution of COXs can be excluded. We also took into account that NDGA, baicalein, GA and di-GA are powerful radical scavengers and antioxidants (Sohi *et al*, 2003; Floriano-Sanchez *et al*, 2006). In case LEC gaps were induced by radicals, gap formation should be inhibited by radical scavengers. To test this possibility, we analysed the efficacy of four *bona fide* ROS scavengers. In particular, we used mannitol, which scavenges the OH^•^ radical; probucol, which is an effective inhibitor of lipid peroxidation; catalase, which is an H_2_O_2_ catabolising enzyme; and carboxy-PTIO, which scavenges the NO^•^ radical. These scavengers did not prevent LEC gap formation. Therefore, MCF-7-induced gap formation was independent of a potential radical involvement.

Finally, we tested whether isolated GA and RV inhibited LEC gap formation. Whereas 50 *μ*M RV inhibited gap size by ∼25%, 80 *μ*M GA was ineffective. Therefore, GA did not affect cell migration, which was in contrast to a galloyl glucose derivate that inhibited tube formation of human microvessel endothelial cells (Lee *et al*, 2004). Methyl gallate influences 5-LOX (Kim *et al*, 2006) and GA may also inhibit this enzyme. However, 5-LOX did not contribute to LEC gap formation, because 100 *μ*M caffeic acid did not reduce gap size (data not shown). This indicated that RV, but not GA, was the inhibitory principle being improved by the higher complex structure of di-GA.

In summary, di-GA dose-dependently inhibited LEC gap formation with an efficiency similar to that of NDGA. The strong anti-invasive property of di-GA is apparently due to the novel chemical structure of the compound, but not due to the GA residues, and only in part due to RV.

## Discussion

Gallic acid is a polyhydroxylated phenol previously known to scavenge radicals, inhibit RR, COXs, LOXs, arrest cell cycle and induce apoptosis (Ha *et al*, 2004; Faried *et al*, 2007; Hsu *et al*, 2007; Madlener *et al*, 2007).

Here we tested a novel synthetic GA derivate, di-GA, assuming that this compound may exhibit superior activity than GA itself. In fact, the pro-apoptotic property of 10 *μ*M di-GA exceeded that of 20 *μ*M GA by four-fold. Thus, an additional pro-apoptotic mechanism, apart from two galloyl residues, contributed to cell death especially at low concentrations. This is of particular interest because such concentrations can be achieved in humans. The RV backbone, to which the galloyl residues are connected, may be responsible for the additive effect, because RV was previously reported to induce apoptosis in HL-60 cells (Horvath *et al*, 2006). The apoptotic activity of di-GA was much higher than the reported RV activity (50 *μ*M RV induced 50% apoptosis in HL-60), but the apoptotic activity of the RV derivative, 3,3′,4,4′,5,5′-hexahydroxystilbene (M8) was even higher than that of di-GA (Horvath *et al*, 2006). In contrast, another RV derivative with anti-neoplastic properties, *N*-hydroxy-*N*′-(3,4,5-trimethoxyphenyl)-3,4,5-trimethoxy-benzamidine (KITC), induced HL-60 apoptosis less efficiently (Saiko *et al*, 2007). Digalloyl-resveratrol triggered apoptosis through the caspase 3 pathway yet independent of p53, because HL-60 cells are p53 deficient (Biroccio *et al*, 1999). Because more than 50% of all cancer types harbour a defective p53 pathway, which is detrimental to successful therapeutic treatment, compounds that exert anticancer activity independent of p53 are of particular interest for clinical applications.

Another prominent anticancer property of therapeutic drugs is to arrest the cell cycle. This can be achieved by blocking distinct mechanisms such as cell-cycle regulators or enzymes involved in DNA-replicative processes etc. Here we show that di-GA inhibited cell proliferation 10-fold more efficiently than GA (Madlener *et al*, 2007). This again suggests that the RV backbone synergised with the two galloyl residues. Similar to GA, di-GA also inhibited HL-60 cell cycle in G1 (Madlener *et al*, 2007). Resveratrol and its analogue M8 were shown to inhibit the cell cycle in S phase (Ragione *et al*, 1998; Horvath *et al*, 2006) and, therefore, the G1-inhibitory effect of the GA moieties was dominant over that of the RV backbone in the di-GA molecule. Interestingly, also KITC inhibited the HL-60 cell cycle in G1 phase (Saiko *et al*, 2007). Digalloyl-resveratrol caused cell-cycle arrest by four independent mechanisms:

Di-GA downregulated cyclin D1 and thus presumably inhibited Cdk4 and/or Cdk6. Cyclin D1 was identified as the Prad 1 oncogene, which is overexpressed in many types of cancer (Lingfei *et al*, 1998; Alao, 2007). Therefore, suppression of cyclin D1 is a relevant target to combat cancer.
Di-GA induced p21^Cip/Waf^ and, therefore, affected Cdk2. Both Cdk2- and Cdk4-activity are mandatory for G1-S transit. Hence, blocking Cdk4 and Cdk2 inhibits cell division. p21^Cip/Waf^ upregulation was independent of p53, because HL-60 cells are p53 deficient. Consistent with reports that p21^Cip/Waf^ is also induced by the MEK–Erk pathway (Facchinetti *et al*, 2004; Park *et al*, 2004), we found that di-GA triggered Erk1(p44Thr202)-phosphorylation within 30 min and MEK1(Ser217)-phosphorylation within 2 h. Further, Erk2(p42Tyr204)-phosphorylation occurred at 4 h, which was simultaneous with p21^Cip/Waf^-induction.
Di-GA stabilised Cdc25A by Ser17 phosphorylation and forced cells through S and M phase. In consequence, ∼90% of the cells accumulated in the following G1 phase due to cyclin D1 suppression and p21^Cip/Waf^ induction. This may have resulted in replicative stress because after 24 h of di-GA treatment Chk2 became activated, which was paralleled by Cdc25A protein degradation. A similar effect was observed on heat shock treatment, which also induces the ATM–Chk2 pathway resulting in the degradation of Cdc25A (Madlener *et al*, 2009). In contrast, Agarwal *et al* (2006) observed an almost immediate Cdc25ASer17 phosphorylation and Chk2 activation on treatment of DU145 cells with GA that was not accompanied by degradation of Cdc25A.
Similar to GA, di-GA inhibited RR most probably by chelating the tyrosyl radical that is required for RR activity (Madlener *et al*, 2007). Resveratrol inhibits RR through a similar mechanism (Fontecave *et al*, 1998). At 5 *μ*M di-GA inhibited 50% of dCTP synthesis, whereas it was reported that 50 *μ*M GA did not inhibit dCTP synthesis whatsoever (Madlener *et al*, 2007). Digalloyl-resveratrol inhibited dCTP synthesis also several-fold more efficiently than RV (Horvath *et al*, 2005). This indicated that the galloyl residues synergised with the RV backbone to inhibit DNA replication.

It has been shown that MCF-7 cells induce gap formation into arterial endothelial cell layers by virtue of 12(*S*)-HETE secretion, which is generated by LOXs metabolising arachidonic acid (Kudryavtsev *et al*, 2005; Uchide *et al*, 2007). Gap formation was due to LEC migration (retraction) but not due to apoptosis of LECs, which was evidenced by microscopic time-laps movies (not shown) and by berberine-mediated inhibition of migration (Ho *et al*, 2009; Tsang *et al*, 2009). We extended this cell system using a three-dimensional co-culture model consisting of MCF-7 spheroids and telomerase-immortalised primary human LECs (Schoppmann *et al*, 2004), because this closely resembles ductal breast cancer bulks intruding the lymphatic vasculature. We showed that MCF-7-triggered lymphendothelial gap formation could be reduced to 40% by NDGA, which is a potent inhibitor of 12/15-LOXs but also a radical scavenger. Several gallate derivates are known to inhibit LOXs (Christow *et al*, 1991; Ha *et al*, 2004; Kim *et al*, 2006), to scavenge radicals (Whang *et al*, 2005) and to inhibit COX (Madlener *et al*, 2007; Kim *et al*, 2006). However, neither radicals nor COXs contributed to gap formation. Hence, baicalein- and NDGA-mediated inhibition supports the notion that at least 50–60% of gap formation was due to 12(*S*)-HETE generating LOX activity. The property of di-GA that reduced LEC migration was similar to that of NDGA. Also the tube formation of human microvessel endothelial cells, which was inhibited by a galloyl glucose derivate, was most likely due to the inhibition of cell migration (Lee *et al*, 2004). Because 12/15-LOX contributes to angiogenesis (Nie *et al*, 2000, 2006; Rose and Connolly, 2000) and tumour metastasis (Liu *et al*, 1996; Jankun *et al*, 2006), di-GA may prevent neo-vascularisation of tumours as well as infiltration of cancer cells into the lymphatic vasculature. Another derivate, galloyl glucose, blocked HT-1080 tumour invasion through gelatin by inhibiting matrix metalloprotease-2 (MMP-2) and MMP-9 (Ata *et al*, 1996). In our system, specific inhibition of MMP-2 and MMP-9 with cell permeable small molecules exhibited only a weak effect on MCF-7-mediated gap formation into LEC layers (data not shown). Interestingly, 80 *μ*M GA did not decrease lymphendothelial gap formation whereas 50 *μ*M RV inhibited gap formation by 25% evidencing that the principal inhibitory activity was contributed by RV and that the superior activity of di-GA was not the sum of RV plus GA, but a new property of its own.

This is analogous to the observation that the RV derivate M8 exhibits not only improved but even new anti-neoplastic properties. In particular, M8 inhibits ROCK1 expression in contrast to RV, which even induces ROCK1 protein levels (Paulitschke *et al*, 2009). ROCK1 supports migration, invasivity and lymph node metastasis of melanoma cells. M8 inhibits melanoma lymph node metastasis in an scid mouse model by ∼50% at a concentration that is comparable to 50 *μ*M used *in vitro* (Paulitschke *et al*, 2009). Interestingly, LEC gaps induced by melanoma spheroids could not be inhibited by NDGA or baicalein suggesting that different cancer types invade the lymphatic vasculature by a mechanism different of LOX. In addition to the effects described above, RV and M8 are shown to inhibit NF-*κ*B (Holmes-McNary and Baldwin, 2000; Horvath *et al*, 2006). In preliminary investigations we found that specific inhibition of NF-*κ*B by small molecules significantly attenuated LEC gap formation (data not shown). Whether di-GA affects ROCK1 expression and/or NF-*κ*B translocation remains to be established. DMU-212 (3,4,5,4′-tetramethoxystilbene) is another RV derivate that exerts strong anti-neoplastic effects in breast carcinoma cells by tubulin polymerisation, which is a mechanism not induced by RV (Ma *et al*, 2008). Other approaches focus on RV analogues with improved cellular uptake properties such as a triacetate form of RV or vineatrol that both retain the anti-neoplastic properties of RV (Colin *et al*, 2009).

In conclusion, we describe three distinct anticancer effects of di-GA: the induction of apoptosis, the inhibition of cell division and the inhibition of gap formation into lymphendothelial layers. Further, we provide mechanistic explanations for the effect of di-GA on apoptosis and cell cycle. For gap formation, we show the affection of cell motility; however, an exact mechanism awaits elucidation.

## Figures and Tables

**Figure 1:**
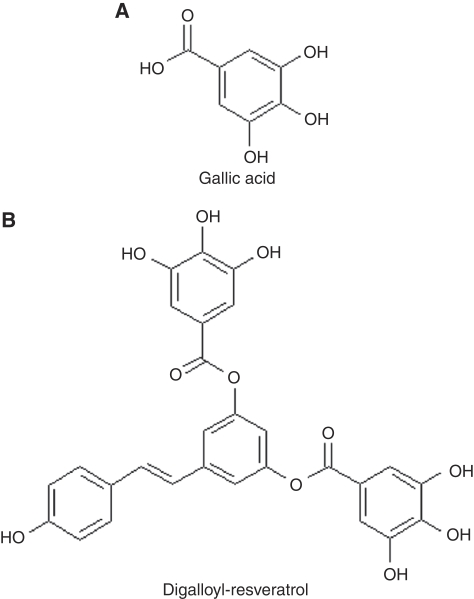
Chemical structures of (**A**) gallic acid (GA) and (**B**) digalloyl-resveratrol (di-GA).

**Table 1:** Concentrations of GA and di-GA that inhibit proliferation of HL-60 cells by 50%

	**I_p_C_50 (24 h)_ (*μ*M)**	**I_p_C_50 (48 h)_ (*μ*M)**
GA	21	24
Di-GA	4	2
